# The Tumor Immune Microenvironment in Clear Cell Renal Cell Carcinoma

**DOI:** 10.3390/ijms24097946

**Published:** 2023-04-27

**Authors:** Cesar U. Monjaras-Avila, Ana C. Lorenzo-Leal, Ana C. Luque-Badillo, Ninadh D’Costa, Claudia Chavez-Muñoz, Horacio Bach

**Affiliations:** 1 Vancouver Prostate Centre, Department of Urologic Sciences, University of British Columbia, 2635 Laurel Street, Vancouver, BC V6H 3Z6, Canada; 2 Division of Infectious Diseases, Department of Medicine, University of British Columbia, 2660 Oak Street, Vancouver, BC V6H 3Z6, Canada

**Keywords:** ccRCC, clear cell renal cell carcinoma, mccRCC, metastatic clear cell renal cell carcinoma, TIME, tumor immune microenvironment, renal cancer

## Abstract

Clear cell renal cell carcinoma (ccRCC) is a type of kidney cancer that arises from the cells lining the tubes of the kidney. The tumor immune microenvironment (TIME) of ccRCC is a complex interplay of various immune cells, cytokines, and signaling pathways. One of the critical features of the ccRCC TIME is the presence of infiltrating immune cells, including T cells, B cells, natural killer cells, dendritic cells, and myeloid-derived suppressor cells. Among these cells, CD8+ T cells are particularly important in controlling tumor growth by recognizing and killing cancer cells. However, the TIME of ccRCC is also characterized by an immunosuppressive environment that hinders the function of immune cells. Several mechanisms contribute to the immunosuppressive nature of the ccRCC TIME. For instance, ccRCC cells produce cytokines such as interleukin-10 (IL-10) and transforming growth factor-beta (TGF-β), which suppress immune cell activation and promote the differentiation of regulatory T cells (Tregs). Tregs, in turn, dampen the activity of effector T cells and promote tumor growth. In addition, ccRCC cells can express programmed death-ligand 1 (PD-L1), which interacts with the programmed cell death protein 1 (PD-1) receptor on T cells to inhibit their function. In addition, other immune checkpoint proteins, such as cytotoxic T-lymphocyte-associated protein 4 (CTLA-4) and lymphocyte activation gene 3 (*LAG-3*), also contribute to the immunosuppressive milieu of the ccRCC TIME. Finally, the hypoxic and nutrient-poor microenvironment of ccRCC can stimulate the production of immunosuppressive metabolites, such as adenosine and kynurenine, which further impair the function of immune cells. Understanding the complex interplay between tumor cells and the immune system in the ccRCC TIME is crucial for developing effective immunotherapies to treat this disease.

## 1. Introduction

Cancer is a complex disease characterized by the uncontrolled proliferation and spread of abnormal cells [1]. Over the years, it has become increasingly clear that the tumor microenvironment (TME) plays a critical role in tumor progression and metastasis. The TME comprises various cellular and non-cellular components, including immune cells, stromal cells, the extracellular matrix (ECM), and blood vessels. Recent research has highlighted the importance of the immune system in regulating tumor growth and metastasis, and the tumor immune microenvironment (TIME) has become an area of intense study [2]. Renal cell carcinoma (RCC) is Canada’s sixth most common malignancy, with an annual increase of 4.2% in men and 2.3% in women [3]. Globally, half of RCC patients is diagnosed with the disease before the age of 65 years [4]. RCC is classified into five major groups with four histological subtypes: (1) clear cell renal cell carcinoma (ccRCC) (>75%), (2) papillary (10%), (3) chromophobe (5%), (4) collecting duct (1%), and (5) unclassified RCC (~4%) [5,6,7]. Clear cell renal cell carcinoma (ccRCC) is a type of kidney cancer that arises from the cells lining the tubes of the kidney.

**Figure 1:**
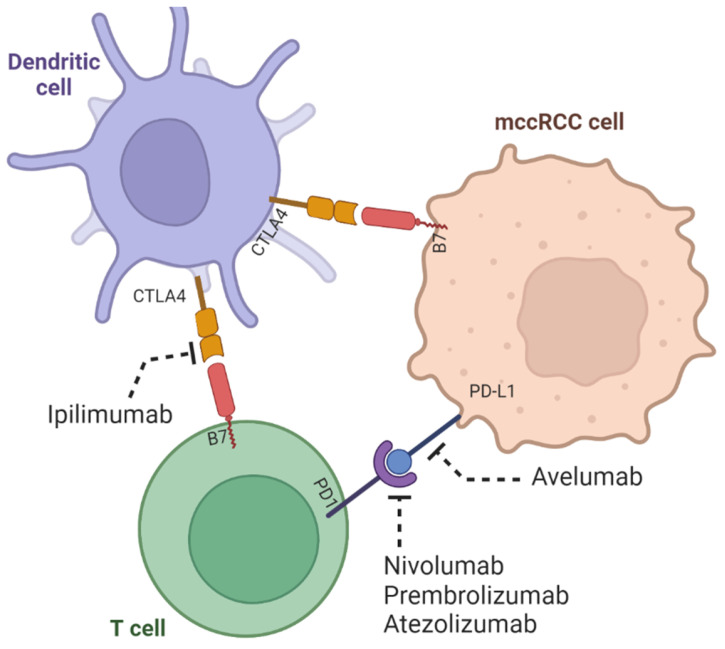
Mechanism of action of immune checkpoint inhibition in mccRCC (Adapted from [11,12]). The figure was created using BioRender.com.

In 1909, Dr. Paul Ehrlich was the first to recognize the protective effect of immunity against cancer [8]. The “immune surveillance” hypothesis was built half a century later, and tumor antigens were discovered [8]. This concept was bolstered by another seminal study on the importance of interferon-γ (IFN-γ) in suppressing tumor growth [9]. Since 1990, cytokines responsible for conferring anti-tumor activity, interferon-α (IFN-α) and interleukin-2 (IL-2), have been used at high doses for the treatment in mccRCC [10]. Unfortunately, both were associated with many side effects; only a small group responded to the treatment [5]. Recent advancements in our knowledge about immune checkpoint receptors, CTLA-4, and PD-1 in cancer immunogenicity and their inhibition led to a paradigm shift in cancer treatment strategies (Figure 1) [11,12].

Despite the characteristics of evading immune surveillance in cancer, many tumors were found to have infiltrated immune cells. Many researchers have addressed this paradox, and there is compelling evidence that immune cells also have tumor-promoting effects [13,14]. Like many other cancers, ccRCC is associated with a complex TIME, and understanding the interplay between the tumor and the immune system is critical for developing new treatment strategies. One of the key features of the ccRCC TIME is the presence of infiltrating immune cells, including T cells, B cells, natural killer (NK) cells, dendritic cells (DC), and myeloid-derived suppressor cells (MDSCs). Among these cells, CD8+ T cells are particularly important in controlling tumor growth by recognizing and killing cancer cells [15]. However, the TIME of ccRCC is also characterized by an immunosuppressive environment that hinders the function of immune cells.

This review provides an overview of the current understanding of the TIME tumor and its role in ccRCC. We also discuss the key components of the TIME in ccRCC, the mechanisms by which immune cells interact with the tumor, the current state of immunotherapy, and the potential future directions of this research.

## 2. Tumor Immune Microenvironment

The TIME comprises various immune and non-immune cells, including T cells, B cells, NK cells, DCs, macrophages, MDSCs, fibroblasts, and endothelial cells. These cells interact with one another and tumor cells through a complex signaling pathway network, leading to a dynamic and ever-changing microenvironment [13,14].

The tumor microenvironment (TME) can be broadly classified into inflamed TME and non-inflamed TME [2,16,17]. The inflamed TME is characterized by immune cells and pro-inflammatory cytokines, which create an immunostimulatory environment that promotes tumor rejection. In contrast, the non-inflamed TME is characterized by the absence or low density of immune cells and the presence of inhibitory factors, creating an immunosuppressive environment that promotes tumor growth.

In ccRCC, the TIME is complex and dynamic, with both immunostimulatory and immunosuppressive factors. The immune infiltrates in ccRCC predominantly comprise T cells, with a high ratio of CD8+ T cells to regulatory T cells (Tregs). In addition, tumor-infiltrating lymphocytes (TILs) are associated with a favorable prognosis in ccRCC. However, ccRCC also has a high density of MDSCs, which can inhibit the anti-tumor immune response [18]. Additionally, ccRCC tumors have an increased expression of immune checkpoint molecules, such as programmed death-ligand 1 (PD-L1), which can inhibit T cell activation and promote tumor immune evasion [19,20].

## 3. Components of TIME in ccRCC

### 3.1. T Cells

CD8+ T cells are critical effectors of the anti-tumor immune response, and their infiltration into the tumor has been associated with improved patient outcomes in several cancer types, including ccRCC [21]. CD4+ T cells are also present in the TIME and can have both pro- and anti-tumor effects depending on their polarization status [22,23]. The presence of T cells in the TIME is thought to reflect ongoing immune surveillance and recognition of tumor antigens.

### 3.2. B Cells

While B cells are not traditionally associated with anti-tumor immunity, recent studies have highlighted their potential role in shaping the TIME in ccRCC [24]. For example, in a study of over 500 ccRCC patients, intra-tumoral B cells were associated with improved overall survival. In addition, B cell infiltration was found to be an independent predictor of survival after adjusting for other prognostic factors [25,26].

### 3.3. Natural Killer Cells

NK cells are immune cells that play an important role in the body’s defense against cancer cells. In ccRCC, NK cells have been shown to have both tumor-promoting and tumor-inhibiting effects. NK cells can promote ccRCC by secreting cytokines and growth factors that support tumor growth and angiogenesis. They can also suppress the activity of other immune cells that could otherwise attack the tumor. NK cells can inhibit ccRCC by directly recognizing and killing cancer cells through several mechanisms, including releasing cytotoxic granules and binding death receptors. NK cells can also enhance the activity of other immune cells, such as T cells, that can help to eliminate the tumor. The balance between the pro-tumor and anti-tumor effects of NK cells in ccRCC likely depends on several factors, including the stage of the disease, the presence of the NK cell subtype, and the composition of the TME. Further research is needed to fully understand the role of NK cells in ccRCC and develop effective strategies for harnessing their anti-tumor activity [27].

### 3.4. Myeloid Cells

Myeloid cells, including macrophages, DCs, and MDSCs, are abundant in the TIME in ccRCC. Macrophages, in particular, have been shown to have both pro- and anti-tumor effects depending on their polarization status [28,29]. In ccRCC, tumor-associated macrophages (TAMs) have been shown to stimulate tumor progression and metastasis by secreting growth factors and cytokines that promote angiogenesis and suppress anti-tumor immunity [30]. Conversely, MDSCs are a heterogeneous population of immature myeloid cells that are thought to suppress T cell function and promote tumor growth [31].

### 3.5. Stromal Cells

In addition to immune cells, fibroblasts produce all components of the ECM in different types of tissues by secreting and creating a diverse range of structural proteins with other characteristics. Each tissue requires a particular ECM tensile strength, which is given by a protein balance between collagen type I and elastin; therefore, fibroblasts secrete a specific amount of each of these proteins in each tissue type [32]. Fibroblasts also have an autocrine secretion; they release cytokines such as TGF-α, IL-6, and IL-1α, promoting inflammatory and fibrotic responses [33]. These cytokines allow fibroblasts to activate macrophages and recruit non-resident immune cells by helping a better extravasation of cells through the endothelium [34]. Fibroblasts also interact with endothelial cells, facilitating the creation of tubes in the connective tissue to create new blood vessels, thereby contributing to angiogenesis. Vascular endothelial growth factor (VEGF) secretion by fibroblasts is the primary mechanism to stimulate angiogenesis through the endothelial cells’ VEGF receptors [35].

**Figure 2:**
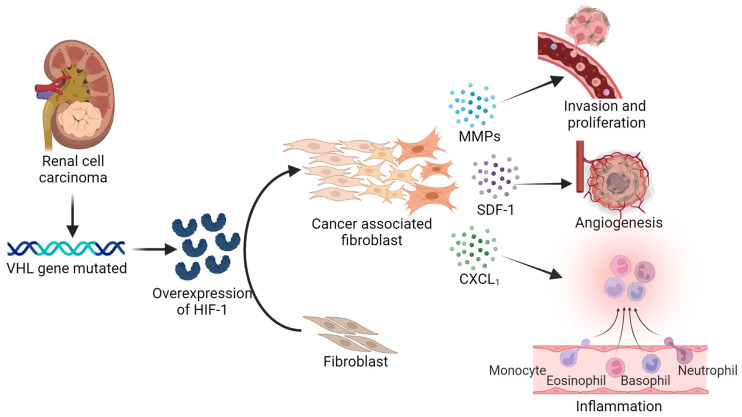
Clear renal cell carcinoma CAFs associated. ccRCC is related to a loss of the *VHL* gene, driving overexpression of HIF-1, and leading to the growth of new blood vessels. CAFs release several factors, such as metalloproteins, that promote invasion and proliferation. CAFs are also involved in inflammation through cytokines and chemokine release. As a result, there is an environment that supports the growth and spread of the tumor. The figure was created using BioRender.com.

Fibroblasts play a crucial role in cancer by regulating the TME and stroma–cancer interactions. They also promote metastasis by remodeling the connective tissue around the tumor, allowing cancer cells to migrate. Once those fibroblasts are part of the TME, they are perpetually activated and called cancer-associated fibroblasts (CAFs). CAFs are a predominant stromal cellular component in most solid tumors, including breast, prostate, and pancreatic cancers. CAF-released factors in the TME play essential roles in tumor development, angiogenesis, metastasis, and therapeutic resistance (Figure 2) [36]. The primary source of CAFs is normal tissue-resident fibroblasts in the tumor that undergo activation. Another source is the endothelial/epithelial-to-mesenchymal transition (EndMT/EMT) process, where endothelial and epithelial cells can turn into CAFs. Bone marrow fibrocytes and mesenchymal stem cells are also sources of CAF. In addition, reports show that other mesenchymal cells, such as pericytes, adipocytes, and vascular smooth muscle cells, could become CAFs under pertinent conditions [37]. Recently, CAFs were subclassified into six subgroups with different characteristics and proportions using various solid cancers. For example, (1) Pan-myCAFs are based on the high levels of activated fibroblast marker ACTA2 and the smooth muscle cell markers MYH11, MCAM, TAGLN, and MYLK; (2) Pan-dCAFs have an elevated expression of collagen genes (COL1A1, COL3A1) and genes associated with ECM remodeling; (3) Pan-iCAF is related to inflammation because of the presentation of a significant level of expression of CFD and C3 genes, and also immunosuppressive factors such as IL-33, CXCL14, and CXCL12; (4) Pan-iCAF-2 has a very high expression of CXCL2, but also a high expression of CXCL1–3, CCL2, IL-6, and IL7; (5) Pan-nCAF is considered to be a normal fibroblast because its hallmark genes for homeostasis are overrepresented; and (6) Pan-pCAF is characterized by the high expression of cell cycle-related genes (BIRC5, TOP2A) [38]. These findings highlight the transcriptome and molecular properties of the CAF subtypes in various cancers.

An important component in TME is CAFs, which can modulate tumor growth by promoting or inhibiting cell proliferation. In addition, CAFs have recently been found to have the benefits of inhibiting tumor progression, making them ideal targets for future therapeutic development. There are, however, very few research studies on CAFs in RCC [39].

Seven key genes of the CAF gene signature have been identified with excellent clinicopathological parameter relationships and prognoses of RCC [40]. In addition, these genes were found to be associated with ECM functions, such as adhesion, collagen synthesis, and cell surface interactions. The CAF-specific gene signatures found were collagen type I alpha 1 chain (COL1A1), collagen type I alpha 2 chain (COL1A2), collagen type V alpha 1 chain (COL5A1), collagen type XVI alpha 1 chain (COL16A1), elastin microfibril interfacer 1 (EMILIN1), lysyl oxidase-like 1 (LOXL1), and lumican (LUM). Interestingly, higher gene expression in groups with stages 1 and 2 was found compared to stages 3 and 4. A high CAF infiltration is related to males <60 years with stages III and IV and grades 3 and 4. An increased number of infiltrated CAFs was associated with a worse prognosis. Therefore, high CAF infiltration in RCC is associated with a poor prognosis and advanced pathological grade and stage. This CAF gene signature may aid in tailored RCC research and treatment and serve as a crucial foundation for future CAF treatment in RCC [40].

Fibroblast activation protein (FAP) is a transmembrane serine protease expressed by CAFs. Cells that express FAP have been described to induce stromal cell-derived factor 1 (SDF-1) expression, promoting tumorigenesis and immunoreaction in different cancer types such as melanoma and pancreatic ductal carcinoma. Furthermore, the expression of this protein could be detected by immunohistochemistry, making FAP a good potential prognostic marker in RCC and several other cancers [41]. 

As mentioned above, ccRCC is related to a loss of the *VHL* gene, driving overexpression of HIF-1 (Figure 2). The result of the HIF-1 increase leads to the expression of factors such as VEGF, SDF-1, and PDGF that recruit and activate normal fibroblasts into CAFs from the TME. Additionally, CAFs appear to play a part in the early stages of the formation of RCC through their association with HIF-1 [37,38,39,40,41,42]. Moreover, CAFs are enriched in high-grade renal tumors, which promotes tumor progression in vitro and in vivo and drug resistance. These CAFs increase kynurenine (Kyn) production by upregulating tryptophan 2, 3-dioxygenase (TDO). A Kyn increase could upregulate the aromatic hydrocarbon receptor (AhR) expression, which leads to the activation of AKT and STAT3 signaling pathways. Therefore, a combination of the AhR inhibitor dimethyl fumarate with chemotherapeutics used in metastatic RCC might reduce renal cancer cells’ ability to spread far [41].

Endothelial cells and pericytes form the blood vessels that supply the tumor with nutrients and oxygen, and their interactions with immune cells and tumor cells play a critical role in the progression of the disease. Tumor angiogenesis is mediated by the endothelial cells responsible for forming new vascularity in the tumor, providing closer access to the blood circulatory system, with increased availability of nutrients and oxygen, triggering cell proliferation and metastasis [43,44]. Overexpression of VEGF will increase the binding with the VEGF tyrosine kinase receptor 1 and 2 (VEGFR1 and VEGFR2) found on the membrane of vascular endothelial cells, leading to endothelial cell activation and stimulating tumor progression [45,46,47,48]. Furthermore, the mutation of *VHL* generates a positive feedback loop in which high levels of VEGF will activate the phosphatidylinositol-3 kinase–AKT pathway, triggering the activation of mTOR, which can stimulate the production of HIFα [45]. This endothelial cell activation releases cytokines that activate CAFs, immune cells, and inflammatory cells [49]. 

Angiogenesis is mediated by the endothelial cells, which form the inner structure of the vessel (arteries, veins, and capillaries) wall. This is a barrier between the blood or lymph flowing inside the vessels and the outer part of the vessel wall. The vascular endothelial cells are in contact with the blood; on the other hand, the lymphatic endothelial cells are in connection with the lymph [50]. Since it has been proven that *VHL* mutations lead to hypoxia condition and angiogenesis in the TME, many treatments target these pathways. The most common therapeutics are VEGF inhibitors, but patients commonly develop drug resistance [51]. For example, the humanized monoclonal antibody bevacizumab is approved as a first-line treatment for ccRCC because it neutralizes VEGF. Studies have shown that in combination with IFN, it resulted in prolonged disease control due to its ability to modulate the vasculature [45,46,47,48,49,50,52]. 

Other therapies for treating angiogenesis in ccRCC have been developed. For example, tyrosine kinase inhibitors (TKIs) do not interact directly with VEGF, but they downregulate the tyrosine kinases activated in the cascade pathways after VEGF phosphorylation. However, the TKIs interacting with multiple signaling pathways rather than VEGF expression have been more effective [45]. That is the case with sunitinib, which can delay tumorigenesis by 6–7 months [53]. However, new TKI therapeutics, such as cabozantinib in combination with nivolumab, an immune checkpoint inhibitor antibody, have improved progression-free survival and overall survival in patients with ccRCC over sunitinib [54]. These treatments could stop the progression of ccRCC in combination with immunotherapy based on preventing hypoxia conditions and angiogenesis in the tumor microenvironment.

## 4. Mechanisms of Immune Cell Interaction with ccRCC and Altered Antigen Presentation

The immune system can recognize and eliminate tumor cells, but tumors have evolved numerous mechanisms to evade immune surveillance and establish an immunosuppressive microenvironment. In ccRCC, several mechanisms have been identified, including those contributing to the evasion of immune surveillance, such as alterations in antigen presentation, induction of immune checkpoint pathways, and recruitment of immunosuppressive cells [55].

Tumor cells express a variety of antigens that can be recognized by T cells. Still, they can also downregulate the expression of major histocompatibility complex (MHC) molecules, which are critical for presenting antigens to T cells. Loss of MHC expression is frequently observed in ccRCC and has been associated with a poorer prognosis [56]. Additionally, tumor cells can upregulate the expression of checkpoint molecules, such as programmed cell death protein 1 (PD-1) and cytotoxic T-lymphocyte-associated protein 4 (CTLA-4), which can inhibit T cell activation and promote immune evasion [57]. Immune checkpoint pathways are a critical mechanism by which tumors evade immune surveillance. Checkpoint molecules, such as PD-1 and CTLA-4, are expressed on T cells and can be activated by ligands expressed on tumor cells or other cells in the TME. These checkpoints can inhibit T cell activation and proliferation, allowing the tumor to evade immune recognition and elimination. Inhibitors of these checkpoints, such as anti-PD-1 and anti-CTLA-4 antibodies, have been approved to treat ccRCC and other cancers and have shown promise in improving patient outcomes [58].

Tumors can recruit various immunosuppressive cells to the TME, including MDSCs, TAMs, and Tregs. These cells can suppress T cell function and promote tumor growth and metastasis. For instance, TAMs, in particular, have been shown to secrete various cytokines and growth factors that promote tumor progression. Moreover, high levels of TAM infiltration in ccRCC have been associated with a poorer prognosis [59]. Conversely, Tregs can suppress T cell activation and promote an immunosuppressive microenvironment by producing TGF-β and IL-10, inhibiting T cell activation and promoting tumor immune evasion [60].

## 5. Therapeutic Implications of the Tumor Immune Microenvironment in ccRCC

The complex interplay between the immune system and the tumor in ccRCC has important implications for developing new treatment strategies. Immunotherapy, which aims to activate the immune system to recognize and eliminate tumor cells, has shown promise in the treatment of ccRCC, and several immune-based therapies have been approved for clinical use.

### 5.1. Immune Checkpoint Inhibitors

Immune checkpoint inhibitors (ICIs) are monoclonal antibodies that block inhibitory molecules on T cells or their ligands on tumor cells, activating the anti-tumor immune response. The most widely studied ICIs in ccRCC are PD-1 and CTLA-4, showing efficacy in the treatment of ccRCC. PD-1 is an inhibitory receptor expressed in T cells, while PD-L1 is a ligand expressed in tumor cells and other immune cells. The binding of PD-1 to PD-L1 inhibits T cell activation and promotes immune evasion [20]. Thus, PD-1/PD-L1 inhibitors can block this interaction, leading to the activation of the anti-tumor immune response. Several PD-1/PD-L1 inhibitors have been approved to treat advanced ccRCC, including nivolumab, pembrolizumab, and atezolizumab.

CTLA-4 is another inhibitory receptor expressed on T cells, which competes with the co-stimulatory receptor CD28 for binding to its ligands on antigen-presenting cells. CTLA-4 inhibitors block this interaction, leading to the activation of the anti-tumor immune response. Examples of this inhibitor include the approved CTLA-4 inhibitor ipilimumab, which has been approved for treating advanced ccRCC.

In a phase 3 clinical trial, treatment with the anti-PD-1 antibody nivolumab significantly improved overall survival compared to everolimus, a standard therapy for advanced ccRCC [61]. Similarly, the combination of nivolumab and ipilimumab has improved progression-free survival and overall survival compared to sunitinib, a standard first-line therapy for advanced ccRCC [62].

In addition, other checkpoint inhibitors, such as antibodies targeting T cell immunoglobulin (VISTA), mucin domain 3 (TIM-3), lymphocyte activation gene 3 (LAG-3), and TIGIT, are also being evaluated in clinical trials for the treatment of ccRCC [63].

VISTA, or the V-domain immunoglobulin suppressor of T cell activation, is a checkpoint molecule expressed on immune cells and some tumor cells. It appears to play a role in suppressing T cell function, allowing tumors to evade the immune system. In addition, studies have found that VISTA expression is elevated in ccRCC tumors and that targeting VISTA with antibodies can enhance the anti-tumor immune response [64].

TIM-3 (T cell immunoglobulin and mucin domain 3) is another checkpoint protein expressed on T cells, NK cells, and other immune cells. TIM-3 regulates immune cell activation and function and has been implicated in tumor immune evasion. Studies have found that TIM-3 expression is elevated in ccRCC tumors, and that blocking TIM-3 can enhance T cell activity and reduce tumor growth in preclinical models [65].

Another checkpoint protein is LAG-3 (lymphocyte activation gene 3), expressed on immune cells, which regulates T cell function. Like VISTA, LAG-3 is upregulated in ccRCC tumors, and blocking it with antibodies has been shown to enhance T cell activity and reduce tumor growth in preclinical models [66].

Lastly, TIGIT (T cell immunoglobulin and ITIM domain) is a checkpoint molecule expressed on NK and T cells. TIGIT interacts with several ligands, including CD155, expressed on some tumor cells. Studies have found that TIGIT expression is elevated in ccRCC tumors and that blocking TIGIT can enhance the anti-tumor immune response [67].

On the other hand, using nanotechnology for medical purposes has emerged as a promising approach for treating ccRCC. However, it is still at the research level. One of the major challenges in the treatment of ccRCC is the delivery of therapeutic agents to the tumor site [68]. Nanoparticles can overcome this challenge by selectively accumulating in tumors due to the enhanced permeability and retention effect.

Several nanomedicine-based therapies have been explored to treat ccRCC, including targeted drug delivery, gene therapy, and immunotherapy [69].

One example of a nanomedicine-based therapy for ccRCC is liposomes, which are spherical structures comprising a lipid bilayer that can encapsulate drugs and other therapeutic agents. In one study, liposomes delivered a small interfering RNA (siRNA) targeting hypoxia-inducible factor 2α (HIF-2α), which is overexpressed in ccRCC. In addition, the liposomes could selectively deliver the siRNA to the tumor site and reduce HIF-2α expression, leading to mouse tumor regression [70].

Another approach involves using nanoparticles as carriers for immunotherapeutic agents such as cytokines or checkpoint inhibitors. For example, one study used nanoparticles to deliver interleukin-2 (IL-2), a cytokine that stimulates the immune system to attack cancer cells. The nanoparticles increased the accumulation of IL-2 in the tumor, leading to improved antitumor activity [71]. Another research group developed polymer proteolysis targeting chimeras as a targeted delivery system for cancer models [72].

In addition to these approaches, ongoing efforts are underway to develop novel nanomedicine-based therapies for ccRCC. For example, researchers are exploring the use of nanorobots, nanoscale devices that can be programmed to target specific cells or tissues to treat ccRCC [73].

Overall, nanomedicine-based approaches show great promise for the treatment of ccRCC, and ongoing research in this field is likely to lead to the development of more effective and targeted therapies in the future. 

### 5.2. Targeted Therapies

Targeted therapies, which aim to inhibit specific signaling pathways that promote tumor growth and survival, have also been developed to treat ccRCC. For example, mitochondrial metabolism has been shown to play a critical role in the progression of ccRCC [74]. In ccRCC, there is an increase in the activation of the hypoxia-inducible factor (HIF) pathway, which leads to the upregulation of glycolysis and the downregulation of mitochondrial metabolism [75]. This shift towards glycolysis, known as the Warburg effect, is thought to provide a metabolic advantage to cancer cells by allowing them to generate energy and biomass rapidly.

However, recent studies have shown that targeting mitochondrial metabolism can disrupt the progression of ccRCC. One of the key regulators of mitochondrial metabolism is the enzyme pyruvate dehydrogenase kinase (PDK), which inhibits the activity of pyruvate dehydrogenase (PDH), a critical enzyme in the mitochondrial metabolism pathway [76]. Inhibition of PDK leads to increased activity of PDH, which promotes mitochondrial metabolism and a decreased reliance on glycolysis.

Studies have shown that inhibition of PDK can suppress the growth of ccRCC cells in vitro and in vivo. Additionally, targeting other components of mitochondrial metabolism, such as the electron transport chain, has also been shown to have anti-tumor effects in ccRCC. These findings suggest that targeting mitochondrial metabolism may be a promising therapeutic approach for the treatment of ccRCC. 

**Figure 3:**
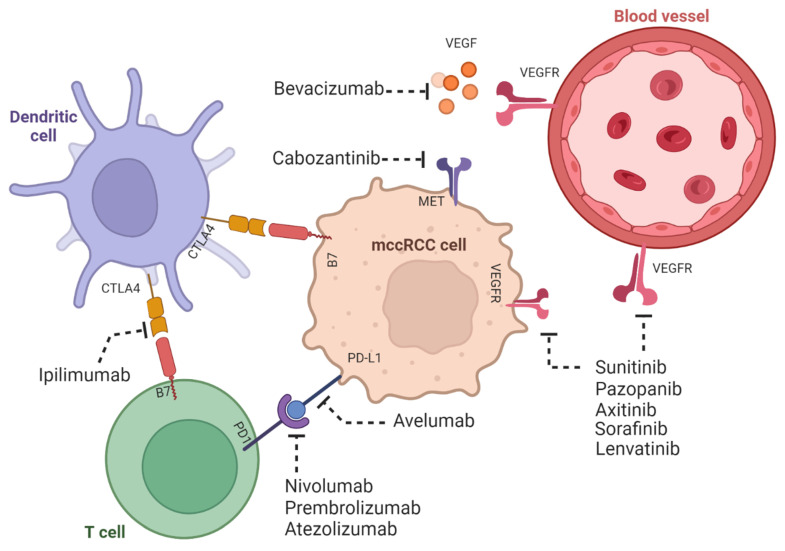
ccRCC tumors are both angiogenic and immunogenic. The mechanisms of the immunogenic response of mccRCC with dendritic and T cells and its effect in angiogenesis. Shown are the therapies involving tyrosine kinase and immune check inhibitors. The figure was created using BioRender.com.

Other therapies include TKIs and inhibitors of the mammalian target of rapamycin (mTOR). While these therapies have shown efficacy in treating ccRCC, they can also have immunosuppressive effects, which may limit their effectiveness in combination with immunotherapy [60]. However, recent studies have shown that combining targeted therapies with immune checkpoint inhibitors may improve patient outcomes (Figure 3) [25,77].

### 5.3. Combination Therapies

Combining immune checkpoint inhibitors with other immunomodulatory agents, such as cytokines, vaccines, or cellular therapies, is a promising strategy for improving the efficacy of immunotherapy in ccRCC. For example, combining the checkpoint inhibitor atezolizumab with the cytokine IL-2 has shown promising results in a phase 1b clinical trial [78]. Similarly, combining the anti-PD-1 antibody pembrolizumab with a cancer vaccine has shown promise in phase 2 clinical trials [79]. In addition, cellular therapies, such as chimeric antigen receptor (CAR) T cells, are also being evaluated as a potential treatment for ccRCC. However, more research is needed to determine their safety and efficacy [80].

### 5.4. Adoptive Cell Therapy

Adoptive cell therapy (ACT) involves transferring ex vivo expanded immune cells, such as T cells or NK cells, back into the patient to enhance the anti-tumor immune response. ACT has shown promising results in treating several cancers, including melanoma and leukemia, but its efficacy in ccRCC is still under investigation [81].

## 6. Future Directions and Conclusions

Despite the promising results of immunotherapy in ccRCC, many challenges remain to be addressed. One major challenge is the identification of predictive biomarkers that can accurately predict responses to immunotherapy. Current biomarkers, such as PD-L1 expression and tumor mutational burden, have limited predictive value, and there is a need for better biomarkers to guide treatment selection.

Another challenge is the development of combination therapies that can enhance the efficacy of immunotherapy. For example, preclinical studies have shown that combining immune checkpoint inhibitors with other immunotherapies, such as cytokines or ACT, can enhance the anti-tumor immune response. Clinical trials are underway to evaluate the safety and efficacy of these combination therapies in ccRCC.

Finally, there is a need for a better understanding of the mechanisms of immune resistance in ccRCC. The tumor immune microenvironment is complex; many factors can contribute to immune resistance. A better understanding of these mechanisms can lead to the development of more effective immunotherapy approaches.

We conclude that clear renal cell carcinoma is a highly immunogenic cancer associated with a complex tumor immune microenvironment. Immune cells and cytokines play a critical role in the progression of the disease, and alterations in the immune microenvironment can contribute to immune evasion and tumor progression. Immune checkpoint inhibitors have shown efficacy in the treatment of ccRCC, and combination therapies that target multiple components of the immune microenvironment are promising strategies for improving patient outcomes.

While significant progress has been made in the development of immunotherapy for ccRCC, there are still many unanswered questions about the mechanisms of immune evasion, understanding mechanisms of immune resistance, and the development of predictive biomarkers for optimal treatment strategies for individual patients. Therefore, further research is needed to identify predictive biomarkers that can guide treatment decisions and to develop new therapeutic approaches that can overcome the challenges of the immunosuppressive microenvironment in ccRCC. Nevertheless, with continued research and development, immunotherapy has the potential to improve the outcomes for patients with ccRCC significantly.

## Data Availability

No data have been generated in this study.
